# Cytoskeletal Regulation of Inflammation and Its Impact on Skin Blistering Disease Epidermolysis Bullosa Acquisita

**DOI:** 10.3390/ijms17071116

**Published:** 2016-07-13

**Authors:** Zlatko Kopecki, Ralf J. Ludwig, Allison J. Cowin

**Affiliations:** 1 Future Industries Institute, Regenerative Medicine, University of South Australia, Mawson Lakes 5095, Adelaide, Australia; allison.cowin@unisa.edu.au; 2 Institute of Experimental Dermatology, University of Lubeck, Lubeck 23562, Germany; Ralf.Ludwig@uksh.de

**Keywords:** actin cytoskeleton, inflammation, skin blistering, epidermolysis bullosa acquisita

## Abstract

Actin remodelling proteins regulate cytoskeletal cell responses and are important in both innate and adaptive immunity. These responses play a major role in providing a fine balance in a cascade of biological events that results in either protective acute inflammation or chronic inflammation that leads to a host of diseases including autoimmune inflammation mediated epidermolysis bullosa acquisita (EBA). This review describes the role of the actin cytoskeleton and in particular the actin remodelling protein called Flightless I (Flii) in regulating cellular inflammatory responses and its subsequent effect on the autoimmune skin blistering disease EBA. It also outlines the potential of an antibody based therapy for decreasing Flii expression in vivo to ameliorate the symptoms associated with EBA.

## 1. Epidermolysis Bullosa Acquisita (EBA)

EBA is a chronic (muco)-cutaneous autoimmune skin blistering condition with an incidence of 0.2–0.5 new cases per million per year [1]. The pathogenicity of EBA is classified by sub-epidermal blistering and tissue bound and circulating autoantibodies at the dermal-epidermal junction against the type VII collagen (COL7) anchoring fibrils [2,3]. EBA patients are classified into two major subtypes: non-inflammatory mechanobullous subtype seen in 33% of patients and the more common inflammatory EBA observed in 66% of patients, which is characterized by cutaneous inflammation and clinically mimics other bullous dermatoses [4,5]. Currently, no controlled clinical trials have been performed on the treatment of EBA and available treatment options involve general immunosuppressive therapy, most commonly colchicine, and/or high doses of systemic glucocorticoids [2], highlighting the need for development of better therapeutic options with targeted modalities specific to EBA [6].

**Figure 1:**
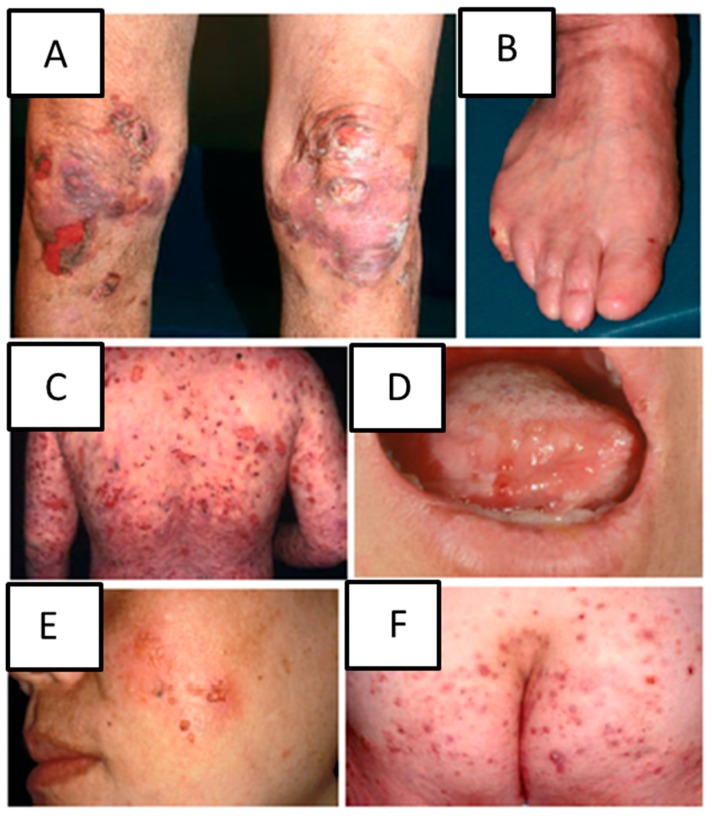
Clinical manifestations of inflammatory epidermal bullosa acquisita (EBA). Blisters and scars on the knee (**A**); loss of toenails (**B**); inflammatory blisters on the back and arms in bullous pemphigoid-like EBA (**C**); oral mucosal involvement in mucous membrane pemphigoid-like EBA (**D**); localized blisters on the cheek in Brunsting-Perry pemphigoid-like EBA (**E**); blisters on the buttock in linear IgA dermatosis-like EBA (**F**). Figure adapted with permission from Kim and Kim 2013 *JEADV* and modified [6].

EBA is diagnosed based on the clinical presentation, detection of tissue bound autoantibodies using direct immunofluorescence and detection of circulating antibodies against COL7 [5]. Patients with inflammatory EBA experience widespread vesiculobullous eruptions involving the trunk, the extremities and skin folds often mimicking other autoimmune bullous dermatoses including bullous pemphigoid, linear IgA, mucous membrane pemphigoid and Brunsting-Perry pemphigoid (Figure 1) [4,6]. EBA belongs to the pemphigoid group of diseases where subepidermal loss of adhesion results in severe blistering [1]. However, many autoimmune diseases manifest with a cutaneous involvement. These include systemic scleroderma, systemic lupus erythematosus and autoimmune skin blistering diseases, e.g., pemphigus/pemphigoid, in which the autoimmune response is directed to antigens, which are structural components of the skin. (Muco)-cutaneous blistering, directly or indirectly caused by the autoantibody binding, is the clinical hallmark of these diseases [7,8].

Pathogenesis of EBA can be broadly divided into three distinct phases including: loss of tolerance to COL7 with subsequent autoantibody production; circulation of the autoantibodies in the bloodstream and antibody induced inflammation and blistering [5]. Studies to date have determined that genetics, T cells, and cytokines all play a critical role in mediating the loss of tolerance towards COL7 and have identified novel potential therapeutic targets for the treatment of EBA [4,9]. The pathogenesis of autoantibody induced tissue injury in EBA is mediated by anti-COL7 antibody binding to COL7, followed by compliment activation, cytokine release mediating neutrophil infiltration, and release of elastase and reactive oxygen species following neutrophil binding to immune complexes [10]. Blister formation activates different signalling pathways aimed at resolving the cutaneous inflammation [4]. In the last decade, the development of different animals models of inflammatory EBA have facilitated the elucidation of the pathogenesis of this autoantibody induced, cell-mediated sub-epidermal disease. These models have included: In vivo antibody transfer induced EBA mouse model and in vivo immunization-induced EBA mouse models reviewed in [11]. Interestingly, studies investigating the mechanisms that underpin blistering and inflammation have highlighted the role of cytoskeletal proteins, particularly Flightless I, during skin blistering [12,13].

## 2. The Actin Cytoskeleton

The actin cytoskeleton is involved in an array of vital cellular functions and has a pronounced influence on many aspects of skin biology [14,15,16]. Despite the structural role of the cytoskeleton, it is highly dynamic and can be rapidly modified to facilitate changes in cell structure during vesicle-organelle transport, cell-cell interactions, cell-extracellular matrix interactions and cell adhesion and motility [17,18,19]. The cytoskeleton comprises a network of filamentous (F)-actin, microtubules, intermediate filaments and stress fibres, all working together to mediate the continual remodelling, assembly and severing needed to generate the mechanical force required for cellular contraction, adhesion and motility [20]. The actin cytoskeleton is a key component required for cellular polarization, force generation, and formation of membrane protrusions, lamellipodia, membrane ruffles and focal adhesions. Actin binding proteins including both structural and adaptor signalling proteins (vinculin, talin, paxillin, α-actinin, Focal Adhesion Kinase (FAK), Src kinase) are involved in regulating actin organisation and polymerisation required for mediation of cell protrusions and migration [21]. Signalling of these proteins results in formation of adhesion sites with downstream signalling to small GTPases of the Rho family regulating the actomyosin dynamics and enabling efficacy and plasticity of leukocyte migration [22]. Integrin mediated cell-matrix adhesions, termed focal complexes develop underneath lamellipodia and are driven by actin polymerisation. These highly dynamic structures develop into elongated focal adhesions associated with necessary contractile stress fibres allowing cell adhesion to the extracellular matrix. Anchoring of the polymerized stress fibres into bundles provides the contractile force required for effective translocation of cell body during cellular migration [23].

## 3. The Role of the Actin Cytoskeleton in Inflammation and Autoimmune Inflammatory Conditions

Recent discoveries have revealed that alterations in actin regulatory and remodelling proteins can results in immune deficiency, autoimmunity and autoinflammatory disease [24]. The deficient or aberrant expression of proteins involved in the regulation of the actin cytoskeleton has increasingly been associated with immunodeficiency and/or autoimmune/autoinflammatory diseases. These regulatory/remodelling actin proteins include actin nucleators (formins and Arp2/3 complex, mDia1), nucleation promoting factors (WASp family, WAVE family, Hemapoetic protein 1), actin stabilizing protein, actin de-polymerising protein (coronin) and actin severing proteins (cofilin, Wdr1) [24]. In relation to inflammation mediated autoimmunity, WASp deficiency results in defects in cellular migration and adhesion, activation and antigen presentation affecting T cells, B cells and dendritic cells of the immune system [25], while WIP deficiency lead to defects in cell chemotaxis, de-granulation and hyperactivity of B cells [24]. In addition, regulation of the actin cytoskeleton is critical for intercellular interactions especially formation of immune synapses and cytotoxic T cell apparatus as well as for inflammatory cell migration within and into the skin [26]. Leucocyte migration from blood through interstitial tissues is essential for mounting of a successful immune response and cell intrinsic and extrinsic factors contribute to proper positioning of the immune cells in the context of special microenvironments [27]. Paracellular and transcellular migration of leucocytes through a process of diapedesis involves number of molecules and mechanism linked to the actin cytoskeleton [28]. Migration of leukocytes from blood to the site of an injury or inflammation in the skin involves a sequence of steps including attachment to the vessel wall, locomotion along the wall to the endothelial borders, transverse migration through the endothelium and the subendothelial basement membrane, and migration through the interstitial tissue [28]. Several endothelial molecules including junctional adhesion molecules, endothelial cell selective adhesion molecules and regulators of endothelial tight junctions have been identified to control transendothelial migration of leukocytes [28]. Additionally, leukocyte perivascular extravasation, migration into the site of tissue injury and adhesion to the extracellular matrix is governed by the polarisation of the actin cytoskeleton [27].

Actin cytoskeletal regulation has also been shown to influence the function of other inflammatory cells including mast cells, dendritic cells, neutrophils and macrophages. Actin cytoskeletal dynamics, including actin filament organisation and distribution of the microtubules in mast cells is governed by GTP-binding proteins [29]. In addition, integrity of the dendritic cell actin cytoskeleton is vital for T cell priming and adhesion during immunological synapse formation [30] while neutrophil contractile forces and cytoskeletal dynamics play an active biophysical role during transmigration through endothelial cell-cell junctions [31]. In context of skin autoimmune inflammatory disease, chemotaxis of macrophages and co-ordinated movement of keratinocytes and activated fibroblasts is directly relevant to the functioning and dynamics of the actin cytoskeleton, including actin control of cell polymerisation, migration and contractibility [32].

## 4. The Gelsolin Family of Actin Remodelling Proteins

The dynamic remodelling of the cytoskeleton is facilitated by the gelsolin family of remodelling proteins which includes gelsolin, villin, adseverin, capG, advillin, supervillin and Flightless I [33]. Gelsolin has been shown to play a role in inflammation and specially inflammatory autoimmune disease, with studies suggesting potential clinical applications for plasma gelsolin in diagnosis and disease activity evaluation as patients with systemic lupus erythematosus (SLE) and rheumatoid arthritis (RA) have significantly decreased plasma gelsolin levels compared to healthy controls [34,35]. Inhibition of the FAK, a non-receptor tyrosine kinase involved in cytoskeleton remodelling and formation and disassembly of cell adhesion structures prevents blister formation in a mouse model of autoimmune skin blistering disease pemphigus vulgaris [36]. Leucine Rich Repeat Flightless Interacting Protein 2 (LRRFlp2) has recently been shown to regulate NLRP3 inflammasome activation in a Flightless I (Flii)-dependant manner [37]. Flii has been shown to control inflammasome activation by way of direct blocking of caspase-1 and caspase-11 and by modulating their subcellular localisation while LRRFlp2 was found to enhance the interaction between Flii and caspase-1 therefore facilitating the inhibitory effect of Flii on caspase-1 activation and subsequent inflammasome activity [37]. Furthermore, silencing of Flii abrogated the inhibitory effect of LRRFlp2 on NLRP3 inflammasome [37] suggesting that an intricate balance of these actin associated cytoskeletal protein is vital in regulation of cellular inflammatory responses, which may have implications in many kinds of diseases including autoimmune disorders. These studies suggest an important cytoskeletal involvement in autoimmune skin conditions. Gelsolin is secreted into plasma to severe the actin filaments that have been released into the circulation following injury and cell necrosis using its gelsolin domain [38,39,40]. Plasma gelsolin is able to inactivate Pathogen-Associated Molecular Pattern (PAMPs) molecules, like Lipopolysaccharide (LPS) and Lipoteichoic acid (LTA), resulting in decreased TLR-mediated NF-κB activity [41,42] and a potential protective role for plasma gelsolin against inflammation.

## 5. Gelsolin Family Member: Flightless I (Flii)

**Figure 2:**
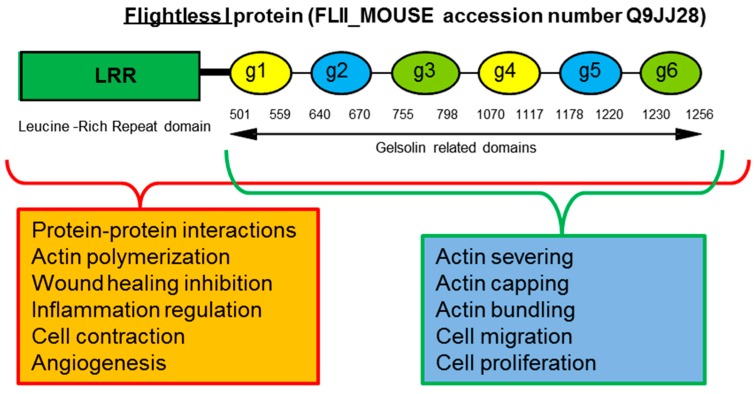
Schematic diagram of Flightless protein structure. Flightless protein consists of Gelsolin segmental repeats (g1–g6) in Gelolin related domain and unique Leucine Rich Repeat Domain (LRR). Each domain has associated cellular functions.

Flii is important in multiple intracellular and extracellular processes. It is a highly conserved multifunctional protein possessing an unique structure, containing 6 gelsolin domains and an additional 11 tandem repeats of a 23-amino acid Leucine Rich Repeat (LRR) motif not present in other family members [43]. Sequence analysis has revealed that Flii retains the gelsolin-like actin binding surfaces but it is missing the calcium binding sites from its gelsolin domains 1–4, however the functional effects of this finding is still unknown [44]. The specificity of its structure allows Flii to use the gelsolin domain to bind and remodel (via severing, capping, and bundling) cytoplasmic actin monomers (G-actin) and actin filaments (F-actin) and it possesses F-actin severing ability [45,46]. Unlike other members of the gelsolin family which enhance actin polymerisation, Flii inhibits actin polymerisation [47] and associates with focal adhesions inhibiting their turnover in a Rac1 dependant manner [48]. Unique specificity of its LRR domain allows Flii to interact with multiple signalling and structural proteins [49] (Figure 2). The bipartite domain structure of Flii provides capacity for it to transduce cell signalling events into remodelling of the actin cytoskeleton and Flii has been proposed to be involved in a variety of signalling pathways [43,45,50,51].

Flii is found in the nucleus, cytosol, lysosomes and like gelsolin is also a secreted protein [38,39,40,52]. Secreted Flii has been detected in human plasma [52], and acute and chronic human wound fluids [52,53]. Flii is constitutively secreted by both fibroblasts and macrophages and this this secretion is increased in response to scratch wounding in fibroblasts and LPS-activated macrophages via a late endosome/lysosome pathway regulated by Rab7 and Stx11 [54]. Secreted Flii protein also binds LPS and has the ability to alter macrophage activation both intracellularly and extracellularly, leading to increased TNF-α cytokine secretion and altered inflammatory responses [52,54] suggesting that Flii upregulation in response to wounding may be directed towards regulating inflammatory cellular responses with unfortunate consequences on healing of wounded area. Flii also binds to proteins other than actin, both in the cytoplasm and in the nucleus, as well as outside the cell. It is sequestered in the cytosol by the active form of the calmodulin-dependent protein kinase type II (CaMK-II) protein [55]. Within the nucleus it binds to a variety of coactivator complexes and to nuclear hormone receptor molecules thereby mediating changes in transcription [56,57,58,59]. Flii, therefore, may provide a link between cell signalling pathways and actin-dependent morphogenetic processes such as proliferation, migration, and adhesion [60,61], all of which influence outcomes of inflammation mediated autoimmune diseases.

## 6. Flii Regulation of the Inflammatory Response

Autoimmune disease develops as a response of parallel mechanisms that relate to the presence of auto-reactive immune cell subsets and loss of immunological tolerance [62]. A key role of the immune system, linking the innate immune responses and adaptive immunity are the germ line encoded receptors, termed pattern recognition receptors (PRRs) [63]. These receptors are present on a variety of immune cells and function to recognize both pathogen-associated molecular patterns (PAMPs), such as bacterial lipopolysaccharide (LPS), and damage-associated molecular patterns (DAMPs), including endogenous danger signals from dead and dying cells [63]. Toll-like receptors (TLRs) are the are the best characterized PRRs, and the triggering of TLRs upon ligand binding results in signaling events that lead to the expression of immune response genes, including inflammatory cytokines, stimulatory immune cytokines and chemokines, which augment the killing of pathogens and initiates the process of developing acquired immunity [64]. However, data arising from human patients and animal models of autoimmune disease suggest that continuous activation or dysregulation of TLR signaling might contribute to the pathogenesis of autoimmunity and tissue damage [65]. Given the important role in autoimmunity TLRs and their signaling pathways have emerged as appealing targets for therapeutics. Understanding how regulators of the actin cytoskeleton, like Flii, contribute to TLR signaling and inflammatory responses in autoimmune diseases like, EBA, may be important for development of novel therapeutics for autoimmune disease.

**Figure 3:**
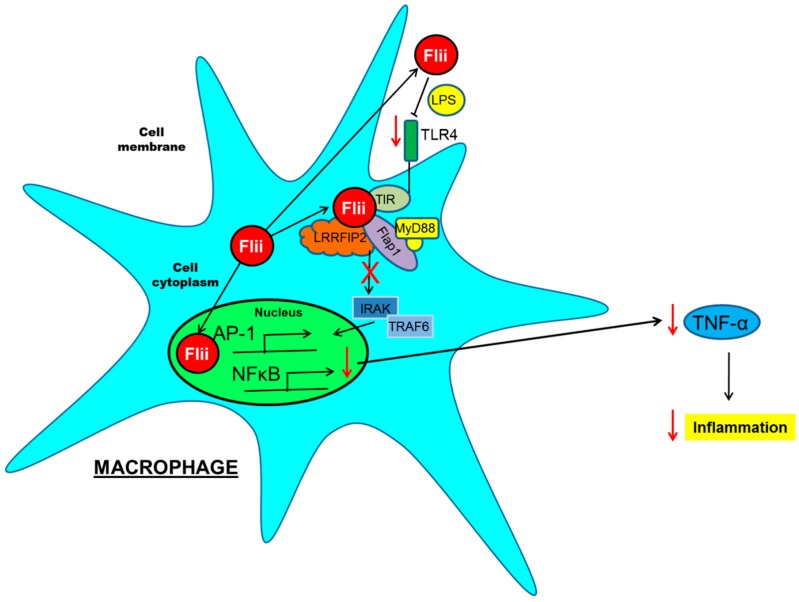
Schematic diagram of Flii involvement in inflammation. Flii is secreted in response to injury/LPS stimulation and current in vitro studies suggest that Flii can dampen TLR inflammatory pathway both extracellularily and intracellularily resulting in decreased TLR signalling, and pro-inflammatory cytokine production. The effects of Flii as a mediator of inflammation in the context of EBA have yet to be investigated. Red arrow pointing down means decreased, and red cross means inhibited.

Flii down-regulates IL-1/TLR4 signalling of Toll-like receptor (TLR) pathways [61] by interacting directly with MyD88, an intracellular adaptor protein immediately downstream of most TLRs [61,64]. TLRs are key innate immune receptors that alert the immune system to tissue damage and mediate the inflammatory response [66]. MyD88 functions to recruit multiple proteins to precisely control signal transduction. A novel switch-on, turn-off mechanism of cytoskeletal regulation of TLR signalling involving Flii has been proposed [61,64]. Flii binds to MyD88 and the Toll/IL-1 receptor domain (TIR) of the TLR molecules to regulate activation of NF-κB, which controls DNA transcription and immune responses to infection [61,64]. By looking at Flii interactions with MyD88, its binding strength and competition with other MyD88 binding partners including Flightless Leucine Rich Repeat Associated Protein-1 and -2 (FLAP-1 and FLAP-2) it has been suggested that Flii may regulate TLR functions [61,64]. The temporal and dynamic nature of specific Flii interactions with other proteins of the TLR signalling has further identified Flii as a negative regulator of the TLR4 signalling pathway [61,64,67]. Another role for Flii in the immune system has been illustrated by its regulation of the pro-inflammatory caspases, namely caspase-1 and caspase-11 [68]. Flii modulates the activity and intracellular localization of these caspases, distributing caspases to the actin network at the leading cell front where they cleave and activate pro-inflammatory cytokines, including IL-1β and IL-18 [68]. In addition, silencing of Flii abrogates the inhibitory effect of LRRFlP2 (FLAP-2) on NLRP3 inflammasome activation in macrophages, suggesting a role for Flii as a mediator of inflammation [37]. The function of extracellular Flii has been linked to its LRR domain as it has nearly 50% homology to the extracellular pathogen binding portion of TLR4 and can bind LPS leading to altered macrophage activation and subsequent cytokine production [54] (Figure 3).

## 7. Role of Flightless I (Flii) in the Inflammatory Autoimmune Disease EBA

**Figure 4:**
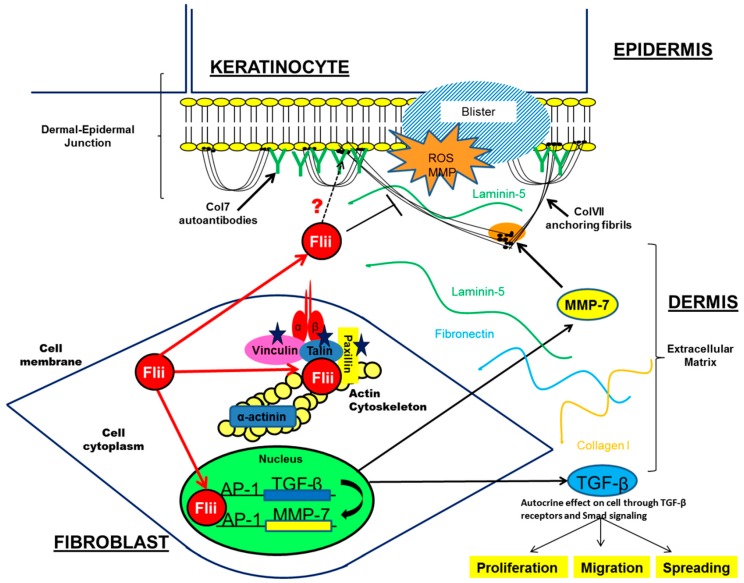
Flii effects on skin blistering in EBA. Intracellularily, Flii associates with intracellular proteins and regulates cell integrin activity and adhesion. In blistered skin, Flii regulates TGF-β signalling which may contribute to increased disadhesion of skin layers. Flii is a secreted protein and has been suggested to be involved in later resolving stages of blister healing. Flii’s possible interaction with Col7 autoantibodies may contribute to increased blistering observed in Flii transgenic mice, identifying Flii as an important modulator of skin blistering in EBA.

Increased expression of Flii leads to thinner and more fragile skin with impaired hemidesmosome structure, decreased COL7 expression at the basement membrane and altered arrangement of COL7 anchoring fibrils [13,69] all features often observed in patients with skin blistering [70]. While Flii over-expressing mice have a normal life expectancy and do not display a blistering phenotype, the effects of Flii on skin architecture were shown to contribute to decreased cell adhesion, irregular hemidesomosome structure, decreased COL7 expression, altered integrin signalling, impaired focal adhesion turnover and increased skin blistering in experimental models of EBA [12,48,69]. In blistered skin, Flii was found to associate with AP-1 proteins, c-fos and c-jun and affect downstream TGF-β signalling [12] suggesting that an interplay between Flii and TGF-β may contribute to increased skin blistering in Flii overexpressing mice. Therefore Flii effects on destabilising the adherence of epidermis to dermis, via its effects on hemidesmosomes, integrin activation, paxillin phosphorylation, TGF-β signalling as well as possible interaction with COL7 autoantibodies, may be a key to increased blistering observed in Flii overexpressing mice (Figure 4). Over expression of Flii resulted in severe blistering post induction of EBA, while in contrast, decreased expression of Flii impaired blister formation in experimental EBA by elevating integrin expression, reducing TGF-β mediated collagen contraction and α-SMA production, reducing Smad signalling and improving COL7 production [12]. These studies point to an important function for Flii in de-stabilising the epidermal-dermal adherence during skin blistering.

Flii is increased in the blistered skin of patients with different subtypes of hereditary epidermolysis bullosa particularly in dystrophic epidermolysis bullosa where a genetic decrease in COL7 expression results in severe skin blistering similar to that observed in EBA [12]. Flii is also detected in human blister wound fluid at similar levels to those observed in acute and chronic wound fluid [52]. Flii expression remains elevated in blister wound fluid for up to three days post blistering [13] and elevated Flii delays the recovery of skin barrier function with decreased Claudin-1 and Claudin-4 expression following blister healing [15]. Taken together these studies suggest that Flii may be involved in the later stages of blister healing and that up regulation of Flii is either related to tissue injury or its effects on regulating inflammation in experimental EBA. Widespread chronic blistering in EBA may further trigger the elevation of pro-inflammatory cytokines through TLR signalling [71] resulting in increased elevation of Flii as observed in blister fluid in order to combat increased inflammation. The effects of Flii on TLR signalling in EBA patients or inflammation mediated skin blistering are yet to be determined. Studies to date suggest that Flii effects on adherence of skin layers may override its pro-resolving anti-inflammatory effects during skin blistering. Nonetheless, these studies demonstrate a relationship between Flii and skin blistering, and suggest that modulation of Flii may improve skin-blistering outcomes.

In contrast, mice with reduced Flii expression exhibit improved rate of wound healing, enhanced epithelial migration and increased wound contraction [72]. Flii effects on collagen secretion, cytokine production and angiogenesis have been described in chronic diabetic and burn injury wounds modes [73,74] and these chronic conditions may be underpinned by Flii effects on tissue inflammation.

## 8. Therapeutic Antibodies for Treatments of Autoimmune Skin Conditions

Emerging treatments for EBA, have focused on different pathophysiologic events associated with this disease including: antibody binding, proinflammatory milieu, blister formation and wound healing [11]. These approaches have identified number of drugs/antibodies and targets aimed at directly or indirectly affecting the clinical efficiency of T cells, B cells, neutrophils as well as mechanisms associated with autoantibody production or wound healing [11]. The use of antibodies to treat autoimmune skin conditions is one of those approaches. Declizumab, a humanized murine monoclonal anti-Tac antibody, showed a good response in one patient with inflammatory EBA [75]. More successful was the use of Rituximab, a chimeric, monoclonal antibody against CD20, a cell surface marker expressed on pre-B cells and mature B cells, which has been used in 12 patients with EBA and shown promising results for patients with either severe and/or relapsing EBA [4]. In addition, a recent study using an immunisation-induced EBA model has shown that use of GM-CSF neutralising antibodies to block inflammation mediated inflammatory cell influx and TLR signalling affected both autoantibody production and skin blistering in a prototypical organ-specific autoimmune disease [76]. Under development is also the use of anti-Hsp90 in a clinical setting, as experimental models of autoimmune bullous diseases have revealed that blockade of Hsp90 underlies the multimodal anti-inflammatory mechanisms of interference with key contributors to auto-immune mediated blister formation [77,78].

**Figure 5:**
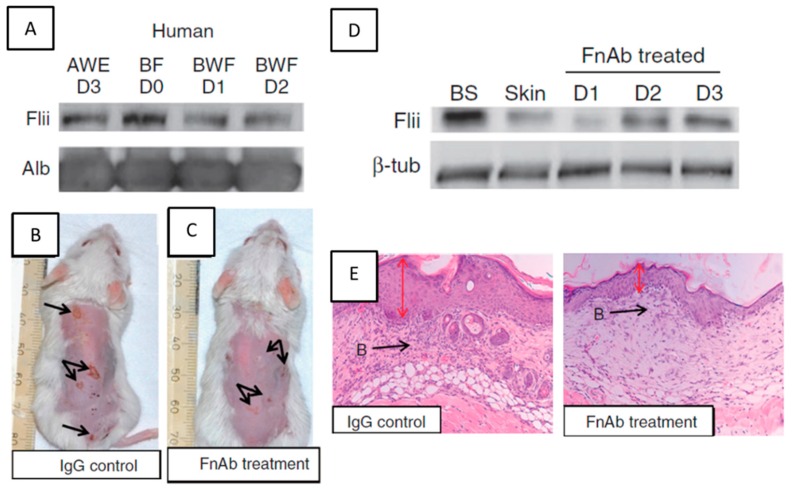
Effect of FnAb therapy on healing of EBA blisters. (**A**) Flii is present in human blister fluid (BF D0) at levels higher then acute wound fluid (AWE D3) and is continually secreted in blister wound fluid over subsequent days 1–2 (BWF D1–D2); (**B**,**C**) FnAb treatment reduces the severity of blistering in a EBA mouse model in wild-type mice; (**D**) Flii is upregulated in blistered skin (BS) compared to unwounded skin and a single FnAb application effectively reduces Flii expression in blistered skin over a period of three days; (**E**) Haematoxylin and Eosin stained blisters at day 16 of the experiment showing decreased inflammatory infiltrate in healing blisters treated with FnAb and thinner epidermis more closely resembling unwounded skin. Figure adapted from Kopecki et al. 2011 *J. Pathol.* and modified [12]. B: Blister as pointed by the black arrow; Red arrow, thickens of epidermis in a healing blister.

A detailed overview of therapeutic options under development has recently been reviewed [11], and here we focus on therapeutic antibody approaches related to actin cytoskeletal regulation of inflammation. Namely, Flii neutralising antibodies have been evaluated in both small and large preclinical animal models of wound healing, burn injury and diabetes and have demonstrated that reducing Flii expression improves both the rate and quality of healing [79]. The use of monoclonal antibody approaches as treatment modalities for improved wound healing have been used previously [80] and in EBA, Flii neutralising antibodies when applied prior to, during, and after blister formation reduces both the incidence and severity of blistering in mouse models [13]. FnAbs, applied topically to the skin, penetrate into the basal epidermis and upper papillary dermis, but are not detected in serum or other organs and do not alter initial neutrophil or macrophage infiltration into the blistered skin [13]. Histological assessment of blister severity showed that treatment of early-stage blisters, or established blisters with FnAb cream reduced the blister severity, improved the rate of healing and restored the skin’s tensile strength toward that of normal skin [13]. The effect of FnAb on the healing of EBA blisters is presented in Figure 5. Future research in this area may lead to novel therapeutic options in a clinical setting.

## 9. Future Directions

The cytoskeleton is intrinsically involved in all aspects of inflammation including inflammatory cell migration, adhesion into affected areas and regulation of cytokine production and cell signalling. Cytoskeletal proteins have multiple roles acting in a time and cell specific manner and can dampen inflammation both inside and outside of the cell and adversely affect the development and/or resolution of blister healing in inflammation mediated autoimmune skin conditions such as EBA. Strategies, including therapeutic antibodies, have recently emerged, and future research will uncover exact mechanisms and pathways affected by some of these pharmacological compounds. Current potent efficacy of some of these compounds, including FnAb, in experimental EBA supports their introduction into clinical settings, giving patients hope for options other than conventional immunosuppressive treatments, however safety profiles or some of these drugs will need to be further investigated or improved. In terms of cytoskeletal regulation of inflammation in autoimmune disease, Flii seems to be leading target for improved wound repair. Understanding the mechanisms that underpin the loss of immune regulation which results in the production of autoimmune disease and imbalance of pro-inflammatory stimuli, anti-inflammatory stimuli and resolving pathways in EBA are clearly required to help improve the chronic morbidity and mortality of inflammation mediated autoimmune disorders.
